# Feasibility and effectiveness of second-line chemotherapy with mitomycin C in patients with advanced penile cancer

**DOI:** 10.3389/fruro.2023.1198980

**Published:** 2024-01-24

**Authors:** Desiree Louise Draeger, Oliver W. Hakenberg

**Affiliations:** Department of Urology, University of Rostock, Rostock, Germany

**Keywords:** penile squamous cell carcinoma, mitomycin c, second-line therapy, palliative, salvage therapy

## Abstract

**Background:**

Triple-drug cisplatin- and taxane-based chemotherapy is the standard treatment for metastatic penile squamous cell cancer (PeSCC), with a moderate response rate of 30% to 38%. Relapse after first-line chemotherapy has a poor prognosis and there is no established second-line treatment. Mitomycin C (MMC) is used as an effective chemotherapy in squamous cell carcinoma of other localities. We therefore used MMC as a single agent for the second-line treatment for patients with advanced PeSCC.

**Methods:**

Nine patients [median age 63 years (range 31 years–81 years)], who, after inguinal and pelvic lymphadenectomy and progression after first-line chemotherapy, received second-line treatment with 20 mg of MMC administered intravenously and weekly, were included in this study. The median number of cycles of MMC was 6 (range 2–12 cycles) and the median cumulative dose was 120 mg absolute (range 40 mg absolute–240 mg absolute). The patients’ toxicity and treatment responses were evaluated, with the latter evaluated using ^18^F-FDG-PET/CT.

**Results:**

Common Terminology Criteria for Adverse Events (CTCAE) grades 3 or 4 thrombocytopenia and grades 2 or 3 leukopenia occurred in all patients, as did anemia. In seven patients, the application interval had to be extended due to thrombocytopenia. Stable disease was achieved in two patients, and all others progressed under treatment. Seven patients died of the disease, with most patients dying 6 months after starting MMC therapy. Of the two patients who responded with disease stabilization, one died of progressive disease 14 months after MMC treatment. The other responding patient has been stable for over 1 year and is still receiving treatment, which he tolerates well, and has a good quality of life.

**Conclusion:**

MMC has only moderate efficacy as a second-line treatment in patients with metastatic PeSCC. With MMC treatment, hematological toxicity is marked.

## Introduction

Penile squamous cell cancer (PeSCC) is a rare disease in industrialized countries, constituting less than 1% of new cancer diagnoses in men but showing a much higher incidence in less-developed countries (1). Advanced PeSCC is associated with a very poor survival due to the lack of effective systemic therapies (2, 3). Triple-drug chemotherapy with cisplatin/taxol/ifosfamide or cisplatin/taxol/5-fluorouracil is the recommended standard treatment for perioperative therapy in lymph-node-positive patients and also for those with systemic disease. These first-line therapies are effective, with a variable response rate reported to be between 30% and 38% (4–7). Survival with progressive disease after first-line chemotherapy is very poor (under 6 months). Currently, there are no known effective second-line treatments.

Patients with good general condition and metastatic disease usually have a strong desire for further treatment. Thus, there is an urgent need for an effective second-line treatment option (8). Although modern antineoplastic treatments such as programmed cell death ligand 1 (PD-L1) or programmed cell death protein 1 (PD-1) inhibitors have been used, there are no reports in the literature demonstrating their significant efficacy in metastatic PeSCC. Mitomycin C (MMC) is an antibiotic drug and was isolated from *Streptomyces caespitosus* in 1958. It has a well-studied activity in many neoplasias, most notably in SCC of the head and neck. It is used as an effective cytotoxic agent albeit, mostly in palliative settings. Its toxicity is mostly due to myelosuppression. Its other reported toxicities are nausea and vomiting, interstitial pneumonia, allergic reactions, and the deterioration of renal function. MMC regimens are commonly used in colorectal, hepatocellular, gastric, breast, esophageal, cervical, and pancreatic cancers, and also in squamous cell cancers of the head and neck, cervix, and anus (9).

We therefore used MMC as cytotoxic monotherapy in patients with progressive PeSCC as a second-line treatment. The aim of this single-arm monocentric trial was to evaluate the potential efficacy of MMC in this situation. To the best of our knowledge, this is the first report of MMC chemotherapy in PeSCC.

## Materials and methods

Nine patients with advanced PeSCC, who were treated at our department between 2018 and 2022, were included in this retrospective analysis. All nine patients had undergone local surgery and inguinal, in addition to pelvic lymphadenectomy bilaterally for nodal involvement. All patients had received first-line chemotherapy with cisplatin-paclitaxel and 5-fluorouracil (eight patients) or cisplatin–paclitaxel and ifosfamide (one patient) adjuvantly and/or for systemic disease. One patient had also received panitumumab as a second-line treatment, but this did not have an effect. Seven patients initially had metastases and had progressed under systemic therapy, and two patients had a metastatic recurrence within several months of undergoing primary therapy.

All patients had progressive disease, a good performance score, and wanted further treatment. All patients were extensively informed about the experimental nature of the treatment and about the fact that this application of MMC was off-label. Thus, informed consent was obtained from all the patients. The retrospective analysis was approved by the Rostock University Medicine internal review board (number A 2021-0258). Since this was an individual palliative therapy about which the participating patients were explicitly informed, this study was not registered in a study register.

The specified exclusion criteria were major surgery or radiotherapy undergone within 4 weeks of the start of therapy, inadequate recovery from previous chemotherapy, concurrent malignancy, mental illness, or a life expectancy of less than 3 months. This was a single-arm retrospective monocentric study.

A 20-mg dose of MMC was administered intravenously, initially at weekly intervals. If hematological toxicity occurred, the treatment intervals were extended to 3 weeks. The application was continued until tumor progression or intolerable side effects occurred. The patients’ response to treatment was monitored every 4–6 MMC doses using ^18^F-FDG-PET/CT, and their clinical chemistry parameters were checked at regular intervals. The demographic and pathological patient data are given in **Table 1**. In regard to comorbidities, the included patients were very heterogeneous. This is partly due to the large age range of the patient cohort. **Table 2** shows the individual characterization of each patient. No patient had received a prior genomic profile examination. Information on human papillomavirus (HPV) or Ki-67 status was reported only very infrequently.

**Table 1:** Baseline characteristics of patients (tumor stages according to the TNM classification of the Union for International Cancer Control, UICC, 8th edition).

Characteristic	Included patients (*n* = 9)
**Mean age**	62 years (SD 25 years, 8 years); range 31 years–81 years
**ECOG score**	2 (0–3)
**Charlson Comorbidity Index (CCI) score**	10 (6–14)
Local T stage	
**pT1**	1
**pT2**	5
**pT3**	3
N stage (initial)	
**pN1**	1
**pN2**	4
**pN3**	4
M stage (initial)	
**cM0**	6
**cM1**	3
**Location of distant metastases ** **Liver** **Lung**	1 2
Grading	
**G1**	0
**G2**	0
**G3**	9
**Course of the disease ** **Initially progressive** **metastatic recurrence**	7 2
**First-line chemotherapy, number of cycles**	5 (1–10)
**Cisplatin/taxol/5-FU**	8
**Cisplatin/taxol/ifosfamide**	1
**Second-line chemotherapy, number of cycles**	3
**Panitumumab**	1
**Number of MMC cycles**	6 (2–18)
**Cumulative average dose**	120 mg (40 mg–360 mg)
**Survival time**	
**Interval from initial diagnosis to death**	12.7 months (6 months–36 months)
**Interval from MMC therapy to death**	5 months (0 months–14 months)

**Table 2:** Treatment parameters of patienmts with second-line MMC treatment.

Characteristics	Patient No. 1	Patient No. 2	Patient No. 3	Patient No. 4	Patient No. 5	Patient No. 6	Patient No. 7	Patient No. 8	Patient No. 9
Age (in years)	31	78	45	67	66	64	52	81	72
ECOG score	1	2	2	2	1	0	2	2	3
Charlson-comorbidity-score (CCI)	12	10	6	10	9	9	6	14	13
HVP status	positiv	n/a	negativ	n/a	n/a	n/a	n/a	n/a	n/a
local T-stage	3	2	3	2	2	2	2	1	3
N-stage (initial)	3	3	3	2	2	2	2	1	3
M-stage (initial)	1	0	1	0	0	0	0	0	1
Location of distant metastases	liver	0	lung	0	0	0	0	0	lung
Grading	3	3	3	3	3	3	3	3	3
Initially progressive	1	0	1	1	1	1	1	0	1
metastatic recurrence	0	1	0	0	0	0	0	1	0
First-line chemotherapy, no of cycles	6	3	6	4	2	6	5	1	10
Type of first-line chemotherapy	cisplatin/taxol/5-FU	cisplatin/taxol/5-FU	cisplatin/taxol/5-FU	cisplatin/taxol/5-FU	cisplatin/taxol/5-FU	cisplatin/taxol/5-FU	cisplatin/taxol/5-FU	cisplatin/taxol/5-FU	cisplatin/taxol/ ifosfamide
Second-line chemotherapy, no of cyclus	0	0	0	0	0	0	0	0	3
pamitumumab	0	0	0	0	0	0	0	0	1
MMC	1	1	1	1	1	1	1	1	1
number of MMC cycles	3	12	2	4	6	18	4	3	3
cumulative average dose in mg	60	240	40	80	120	360	80	60	60
Survival time (death = 0, alive 1)	0	0	0	0	0	1	0	0	0
interval from intial diagnosis to death (in months)	8	36	6	12	8	Still alive	9	11	12
interval from MMC therapy to death (in months)	3	14	0	5	6	Still alive	6	5	1
Adverse Events (grade, CTCAE)	Pancytopenia (4), nausea and vomiting (3)	Pancytopenia (3)	Pancytopenia (4), nausea and vomiting (2)	Pancytopenia (3)	Pancytopenia (3)	Pancytopenia (3)	Pancytopenia (3). nausea and vomiting (2)	Pancytopenia (3)	Pancytopenia (4), stomatitis (3)

n/a, not applicable.

The primary end points were tumor response and toxicity. Toxicity was evaluated in all patients. The overall survival (OS) in this context was defined as the time between the beginning of the second-line MMC treatment and death. The median follow-up was estimated using the reverse Kaplan–Meier method. Univariate Cox proportional hazards models were used to estimate the effect of each predictor on OS. All tests were two-sided and *p*-values under 0.05 were considered significant. All statistical analyses were performed using the statistical software IBM SPSS Statistics 27 (version 2020; IBM Corporation, Armonk, NY, USA). Follow-up data were collected at regular intervals through clinical examination in our outpatient clinic or by phone from outpatient physicians. The Common Terminology Criteria for Adverse Events (CTCAE) Version 5.0 was used to classify toxicity.

## Results

The median patient age was 62 years (range 31–81 years) at the beginning of MMC treatment. The mean Eastern Cooperative Oncology Group Classification (ECOG) score was 2 (0–3), with the majority of patients having an ECOG score of ≥ 2. First-line chemotherapy had been given with a median of five cycles (1–10) until progression occurred. The median follow-up was 6 months (**Table 1**).

The median number of cycles of MMC given was 6 (range 2–12 cycles), and the median cumulative dose was 120 mg (range 40 mg–240 mg). Asymptomatic CTCAE grades 3 or 4 thrombocytopenia was seen in all patients. Similarly, grades 2 or 3 leukopenia and some degree of anemia occurred in all patients. Whether anemia was due to the treatment or the disease, however, could not be determined. Three patients developed transient nausea and vomiting, but only with the first application of MMC. One patient developed transient stomatitis.

Regular PET/CT scanning showed that six patients progressed under MMC treatment. Only two patients achieved stable disease, and this was the best response seen. In all patients, the application interval had to be extended due to thrombocytopenia after the second or third application of MMC. No dose reductions were made.

**Figure 1:**
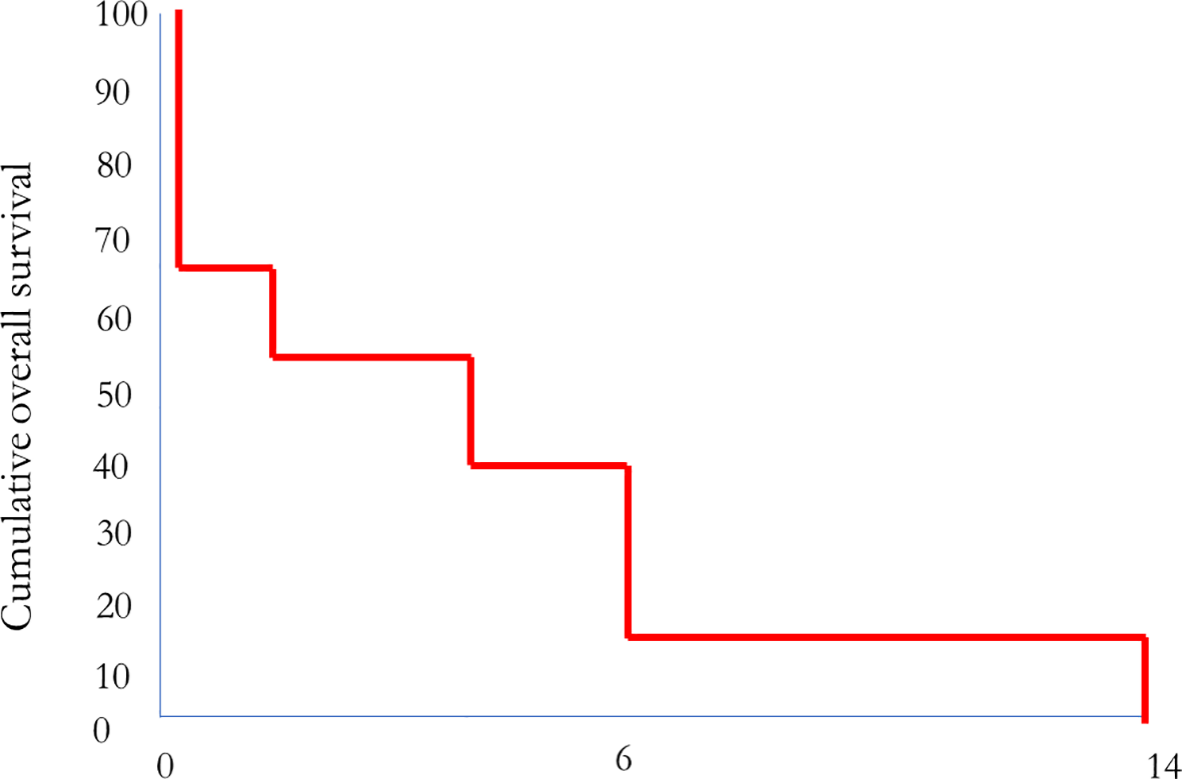
Correlation between MMC treatment and overall survival (*n* = eight patients; one patient is still alive).

Seven patients died of disease, on average, 5 months after starting MMC therapy. Four of these patients had not received any MMC treatment for the 3–12 weeks before death due to the deterioration of their general condition. Two patients died on therapy. One patient with initially stable disease died of disease 14 months after the initiation of MMC treatment (**Table 1**; **Figure 1**). The remaining patient who responded with stable disease is alive 18 months after the start of MMC treatment. This patient has a good quality of life, no toxicity, and receives maintenance MMC treatment.

The treatment response was monitored every 4–6 MMC doses using a ^18^F-FDG-PET/CT scan. In the patients with progressive disease, qualitative and quantitative progression was seen in the PET/CT scan. In the patients with stable disease, there was no numerical decrease in the metastases, but a clear reduction in the signal intensity, with the metastases appearing as low-intensity FDG-positive masses without reliable evidence of residual vitality. In the one patient with long-term response for over 1 year, PET/CT imaging has shown no change in the size of the disease lesions, but also no clear evidence of tumor vitality.

After the third application of MMC, pronounced thrombocytopenia was seen in most patients. Usually, in chemotherapy, bone marrow suppression manifests alongside leukopenia and less commonly with thrombopenia.

## Discussion

Penile squamous carcinoma (PeSCC) is a biologically aggressive disease with rapid invasive growth and early lymphatic metastasis. Although penile cancer is rare, its incidence is increasing, with a 21% increase in the incidence rate seen in the last decade. Due to its absolute rarity, however, there are few large prospective clinical trials providing data to guide the management of locally advanced and metastatic PeSCC. Cisplatin-containing combination chemotherapy regimens are widely regarded as the standard of care (SOC) in this setting. However, with a response rate of only approximately 50%, there is a need to further improve the treatment outcomes. New and efficient therapeutic strategies are needed to improve patient survival rates.

Systemic chemotherapy is used for the neoadjuvant and/or adjuvant treatment of regional lymph node disease in conjunction with lymphadenectomy and also for systemic metastatic disease. In metastatic disease, chemotherapy is mostly palliative (8). The established regimens are cisplatin and taxane based, with the combination of paclitaxel, cisplatin, and 5-fluorouracil used mostly in Europe and that of cisplatin, paclitaxel, and ifosfamide in the United States (4–8, 10). The prognosis in relapsing metastatic disease after primary chemotherapy is poor, and survival is less than 6 months (4). There is no established second-line treatment, although there is an urgent need for it.

Paclitaxel was tested as a second-line treatment in a small phase II study, but the response rate was below 30% and did not achieve any significant increase in survival (8, 11, 12). There have also been reports on the second-line use of gemcitabine in combination with cisplatin, but with similarly poor success (13–15). Tyrosine kinase inhibitors (such as sorafenib and sunitinib) have been reported to be ineffective. In 2018, a phase II study with dacomitinib, a pan-human epidermal growth factor receptor (HER) inhibitor, described a first-line treatment with a remission rate of 32% in 28 patients, but in which there was no second-line application (16). Trials using epidermal growth factor receptor (EGFR)-directed antibodies as a second-line treatment was also unsuccessful (17, 18). Outside of studies, PD-L1 has been used, but no positive results have been published. Cemiplimab, a highly potent, hinge-stabilized human immunoglobulin G4 (IgG4) monoclonal antibody, which blocks PD-L1 and PD-L2 (19), can be used for advanced SCC of other origins. It selectively stimulates the immune system. It increases the function of the T cells (i.e., their proliferation, cytokine release, and cytotoxic activity). Its half-life is 19.2 days (19–22). PD-L1 is upregulated in 40%–60% of PeSCC cases and is correlated with a poor prognosis, making a case for immunotherapy as a treatment for metastatic PeSCC. Currently there are ongoing clinical trials exploring immunotherapy in PeSCC; however, none of these trials is investigating the combination of immunotherapy and chemotherapy. The novel immune checkpoint inhibitor cemiplimab has been approved for locally advanced and metastatic cutaneous squamous cell carcinoma in a palliative setting only. The phase II-EPIC trial evaluates the efficacy and safety of cemiplimab alone or in combination with SOC chemotherapy in patients with locally advanced or metastatic PeSCC, but results are still pending (23). There are some case reports of the use of cemiplimab in platinum-resistant penile carcinoma with a complete response. No toxicity was reported (24, 25).

HPV infections are considered a major etiologic factor. Due to their oncogenes E6 and E7, HPV viruses disrupt the cellular protective regulations based on the p53 and RB regulatory circuits. However, HPV virus material can be detected only in some forms of penile PeSCC. In the most common histologic subtype, classic squamous cell carcinoma, this rate is 30%–50%, whereas in basaloid and warty squamous cell carcinoma it is 80%–100%. These differences point to a different genesis of the various forms of PeSCC. Based on the geographical location, the available literature, and our own data, an HPV prevalence of approximately 50% in PeSCC can currently be assumed worldwide, and the trend is increasing (26, 27). However, the literature data on HPV prevalence and incidence should not be viewed uncritically. In addition to the often inconsistent documentation of the anatomical localization and inconsistent HPV diagnostics, there is a lack of comprehensive data on the HPV status at national and international levels. In many epidemiological studies, the HPV prevalence is derived from local data from other studies, which leads to a great deal of uncertainty and shows that there is still a need for research, especially at national and international levels. It is known from other SCC entities that HPV-associated cancers might more be responsive to immunotherapy (28). Current immunooncological therapeutic approaches target the immune checkpoints, a series of receptor–ligand combinations that regulate the activity of the body’s own immune cells and suppress rejection reactions to the body’s own cells. By expressing correspondingly effective surface molecules, many tumors use these interactions to evade the activity of immune cells. Therapeutic antibodies can interfere here and make tumor cells vulnerable to the immune system again. PD1 and its ligand (PD-L1) are currently among the most promising starting points for urological immunotherapy. It can be assumed that patients with HPV-associated PeSCC might benefit from checkpoint immunotherapy, since these tumors express exogenous antigens (viral oncoproteins) and could be particularly amenable to a reactivated immune system (29). Regardless of the patient’s HPV status, 40%–60% of penile cancers show a high level of PD-L1 expression which, although associated with decreased cancer-specific survival, could nevertheless be a rationale for a checkpoint inhibitor-based treatment approach (3, 24). The predictive value of HPV status and PD-L1 positivity should therefore be investigated.

To improve clinical reliability, further criteria for the histologic examination appear to be necessary. The study situation on molecular biological markers in PeSCC is limited due to the overall small number of cases and most of the retrospective studies having a small number of patients. In addition, these studies were performed using very different methods. If a patient has already had several tumor diseases or has a positive family history of tumor diseases, it can be useful to determine their microsatellite status (MSI) before starting immunotherapy. However, so far there is evidence showing only that microsatellite instability is rare in PeSCC (30, 31). Nevertheless, MSI and the deficient mismatch repair (dMMR) system proteins represent predictive biomarkers for the response to immune checkpoint therapy. Until now, few data related to PD-L1 expression and MSI in PeSCC have been reported (30, 31). Pembrolizumab can be recommended for patients with PeSCC with a high MSI or dMMR. PD-L1 expression and MSI status could represent such potential biomarkers in predicting immunotherapy efficacy in PeSCC. Further clinical trials using immune checkpoint blockade regimes in patients with PD-L1 expression and a MSI-H status may clarify the efficacy of immunotherapy and its possible clinical application in PeSCC (30).

Despite the rarity of the disease, especially in the metastatic stage, prospective clinical trials providing data on the benefit of systemic therapy in patients with PeSCC are urgently needed. Affected patients should, if possible, be included in studies. Various phase II studies to clarify the effectiveness of immunotherapy in PeSCC have recently begun, and these are detailed in **Table 3**:

**Table 3:** Ongoing immunotherapy trials for penile cancer.

NCT03686332	PD-L1 inhibitor atezolizumab ± local radiotherapy
NCT03391479	PD-L1 inhibitor avelumab in patients who are unfit for or progressed on platinum-based chemotherapy
NCT04224740	First-line pembrolizumab in combination with chemotherapy
NCT03774901	Maintenance therapy with avelumab after patient response to first-line chemotherapy
NCT03866382	Nivolumab, ipilimumab, and cabozantinib
NCT03333616/NCT02834013	Nivolumab and ipilimumab
NCT03439085	Durvalumab in combination with a vaccine in HPV-associated cancers

Thus, at present, there remains a dismal prognosis for patients with disease progression after first-line chemotherapy. However, trials focusing on immunotherapy are ongoing, but presently it is unclear what efficacy can be expected.

Regarding second-line chemotherapy with MMC, in our small trial group, two of the MMC patients survived longer than 6 months and gained some benefit from the MMC second-line treatment. One patient died 14 months after the start of MMC treatment, which was a survival period significantly longer than that seen in non-responders. The other patient is doing remarkably well and has now undergone an 18th cycle of MMC.

This corresponds to a response rate of 11% in nine patients. Seven patients died of progressive disease without receiving any benefit from the second-line MMC treatment. Overall, MMC does not seem to be of sufficient efficacy as a second-line treatment (although some patients may benefit from it), but there is no evidence as to which patients might respond. Obviously, the retrospective data evaluation is a limitation of this small study. However, our data indicate that a prospective trial with MMC is most likely not warranted.

The expectations are that some improvement in the treatment options for penile cancer patients after failed first-line chemotherapy, which is urgently needed, may hopefully be achieved through progress with immunotherapeutic options.

## Conclusion

In conclusion, second-line MMC treatment in progressive penile SCC is not a generally effective second-line chemotherapy option. Some patients might benefit from it; however, there are no known prognostic factors that might indicate which patients would benefit from this treatment.

## Data availability statement

The raw data supporting the conclusions of this article will be made available by the authors, without undue reservation.

## Ethics statement

The study was conducted in accordance with the guidelines of the Declaration of Helsinki, and approved by the Institutional Ethics Committee of University Medical Center Rostock. Informed consent was obtained from all participants involved in the study.

## Author contributions

DD collected clinical data and analyzed the data. DD and OH wrote the manuscript. All authors provided critical feedback and helped shape the manuscript. All authors contributed to the article and approved the submitted version.

## References

[1] Siegel RL Miller KD Jemal A. Cancer statitics 2020. CA Cancer J Clin (2020) 70:7–30. doi: 10.3322/caac.21590 31912902

[2] Sonpavde G Pagliaro LC Buonerbe C Dorff TB Lee RJ Di Lorenzo G. Penile Cancer: current therapy and future directions. Ann Oncol (2013) 24:1179–89. doi: 10.1093/annonc/mds635 PMC4047287 23293117

[3] Udager AM Lui T Skala SL Magers MJ McDaniel AS Spratt DE. Frequent PD-L1 expreesion in primary and metastatic penile squamous cell carcinoma: potential opportunities for immunotherapeutic approaches. Ann Oncol (2016) 27:1706–12. doi: 10.1093/annonc/mdw216 PMC4999561 27217541

[4] Nicholson S Hall E Harlang SJ Chester JD Pickering L Barber J. Phase II trial of docetaxel, cisplatin and 5FU chemotherapy in locally advanced and metastatic penis cancer (CRUK/09/001). Br J Cancer (2013) 109(10):2554–9. doi: 10.1038/bjc.2013.620 PMC3833214 24169355

[5] Di Lorenzo G Buonerba C Federico P Perdonà S Aieta M Rescigno P. Cisplatin and 5-fluorouracil in inoperable, stage IV squamous cell carcinoma of the penis. BJU Int (2012) 110(11 Pt B):E661–6. doi: 10.1111/j.1464-410X.2012.11453.x 22958571

[6] Haas GP. Cisplatin, methotrexate and bleomycin for the treatment of carcinoma of the penis: a Southwest Oncology Group study. J Urol 161(6) (1999) p:1823–5. doi: 10.1097/00005392-199906000-00025 10332445

[7] Theodore C Skoneczna I Bodrogi I Leahy M Kerst JM Collette L. A phase II multicentre study of irinotecan (CPT 11) in combination with cisplatin (CDDP) in metastatic or locally advanced penile carcinoma (EORTC PROTOCOL 30992). Ann Oncol (2008) 19(7):1304–7. doi: 10.1093/annonc/mdn149 18417462

[8] Leitlinienprogramm Onkologie (Deutsche Krebsgesellschaft, Deutsche Krebshilfe, AWMF): S3-Leitlinie Diagnostik, Therapie und Nachsorge des Peniskarzinoms, Langversion 1.0, 2020, AWMF Registernummer: 043–042OL. Available at: https://www.leitlinienprogramm-onkologie.de/leitlinien/peniskarzinom/.

[9] Mitomycin Medac. Fachinformation. Rote Liste Service GmbH. Fachinfo-Service Mainzer Landstraße 55. 60329 Frankfurt.

[10] Hakenberg OW Dräger DL Erbersdobler A Naumann CM Jünemann KP Protzel C. The diagnosis and treatment of penile cancer. Dtsch Arztebl Int (2018) 115:646–52. doi: 10.3238/arztebl.2018.0646 PMC6224543 30375327

[11] Di Lorenzo G Federico P Buonerba C Longo N Cartenì G Autorino R. Paclitaxel in pretreated metastatic penile cancer: final results of a phase 2 study. Eur Urol (2011) 60(6):1280–4. doi: 10.1016/j.eururo.2011.08.028 21871710

[12] Wang J Pettawa CA Pagliaro LC. Treatment for metastatic penile cancer after first-line chemotherapy failure: analysis of response and survival outcomes. Urology (2015) 85:1104–10. doi: 10.1016/j.urology.2014.12.049 25819619

[13] Houede N Dupuy L Fléchon A Beuzeboc P Gravis G Laguerre B. Intermediate analysis of a phase II trial assessing gemcitabine and cisplatin in locoregional or metastatic penile squamous cell carcinoma. BJU Int (2016) 117(3):444–9. doi: 10.1111/bju.13054 25601543

[14] Liu JY Li YH Liu ZW Zhang ZL Ye YL Yao K. Intraarterial chemotherapy with gemcitabine and cisplatin in locally advanced or recurrent penile squamous cell carcinoma. Chin J Cancer (2013) 32(11):619–23. doi: 10.5732/cjc.012.10275 PMC3845548 23668929

[15] Power DG Galvin DJ Cuffe S McVey GP PMulholland PJ Farrelly C. Cisplatin and gemcitabine in the management of metastatic penile cancer. Urol Oncol (2009) 27(2):187–90. doi: 10.1016/j.urolonc.2007.10.015 18367122

[16] Necchi A Lo Vullo S Perrone F Raggi D Giannatempo P Calareso G. First-line therapy with dacomitinib, an orally available pan-HER tyrosine kinase inhibitor, for locally advanced or metastatic penile squamous cell carcinoma: results of an open-label, single-arm, single-center, phase 2 study. BJU Int (2018) 121(3):348–56. doi: 10.1111/bju.14013 28921872

[17] Gou HF Li X Qiu M Cheng K Li LH Dong H. Epidermal growth factor receptor (EGFR)-RAS signaling pathway in penile squamous cell carcinoma. PloS One (2013) 8(4):e62175. doi: 10.1371/journal.pone.0062175 23637996 PMC3634795

[18] Wu J Cheng K Yuan L Du Y Li C Chen Y. Recurrent penile squamous cell carcinoma successfully treated with cetuximab, chemotherapy, and radiotherapy. Clin Genitourin Cancer (2016) 14(1):e135–7. doi: 10.1016/j.clgc.2015.10.010 26585838

[19] Product information on LIBTAYO (cepilimab). Available at: https://www.ema.europa.eu/en/documents/product-information/libtayo-epar-product-information_de.pdf.

[20] Ahmed SR Petersen E Patel R Migden MR. Cemiplimab-rwlc as first and only treatment for advanced cutaneous squamous cell carcinoma. Expert Rev Clin Pharmacol (2019) 12(10):947–51. doi: 10.1080/17512433.2019.1665026 31524530

[21] Lee A Duggan S Deeks ED. Cemiplimab: A review in advanced cutaneous squamous cell carcinoma. Drugs (2020) 80(8):813–9. doi: 10.1007/s40265-020-01302-2 32306208

[22] Migden MR Khushalani NI Chang ALS Lewis KD Schmults CD Hernandes-Aya L. Cemiplimab in locally advanced cutaneous squamous cell carcinoma: results from an open-label, phase 2, single-arm trial. Lancet Oncol (2020) 21(2):294–305. doi: 10.1016/S1470-2045(19)30728-4 31952975 PMC7771329

[23] Renninson E Challapalli A Foulstone E Bravo A Soundy A Callaway M. A phase II trial of cemiplimab alone or in combination with standard of care chemotherapy in locally advanced or metastatic penile cancer (EPIC trial). J Clin Oncol (2022) 40(16). supple—ASCO poster session. doi: 10.1200/JCO.2022.40.16_suppl.TPS5094

[24] Steck S Cathomas R Kienle D. Profound and durable responses with PD-1 immune checkpoint inhibitors in patients with metastatic penile squamous cell carcinoma. Curr Problems Cancer: Case Rep (2021) 3:100045. doi: 10.1016/j.cpccr.2020.100045

[25] Denis C Sakalihasan S Frères P Withofs N Sautois B. Cemiplimab for cisplatin resistant metastatic penile cancer. Case Rep Oncol (2021) 14(2):972–6. doi: 10.1159/000517008 PMC8261263 34267641

[26] Buonerba C Pagluica M Vitrone FM Ascione I Elefante I Riccio V. Immunotherapy for penile cancer: Future Science OA 3, FSO 195. (2017). 10.4155/fsoa-2017-0031 PMC5583688 28883996

[27] Hernandez BY Goodman MT Unger ER Steinau M Powers A Lynch CF. Human papillomavirus genotype prevalence in invasive penile cancers from a registry-based United States population. Front Oncol (2014) 4:9. doi: 10.3389/fonc.2014.00009 24551592 PMC3914298

[28] McGregor B Sonpavde G. Immunotherapy for advanced penile cancer—rationale and potential. Nat Rev Urol (2018) 15:721–3. doi: 10.1038/s41585-018-0083-0 30166593

[29] Skeate JG Woodham AW Einstein MH Da Silva DM Kast MW. Current therapeutic vaccination and immunotherapy strategies for HPV-related diseases. Hum Vaccin Immunother (2016) 12:1418–29. doi: 10.1080/21645515.2015.1136039 PMC4964648 26835746

[30] Montella M Sabetta R Ronchi A De Sio M Arcaniolo D De Vita F. Immunotherapy in penile squamous cell carcinoma: present or future? Multi-target analysis of programmed cell death ligand 1 expression and microsatellite instability. Front Med Sec Pathol (2022) 9. doi: 10.3389/fmed.2022.874213 PMC9113025 35592855

[31] Stoehr R Wendler O Giedl J Gaisa NT Richter G Campean V. No evidence of microsatellite instability and loss of mismatch-repair-protein expression in squamous cell carcinoma of the penis. Pathobiology 86:145–51. doi: 10.1159/000495251 30650417

